# Characterization of the bacterial microbiota in different gut and oral compartments of splendid japalure (*Japalura sensu lato)*

**DOI:** 10.1186/s12917-022-03300-w

**Published:** 2022-05-27

**Authors:** Zhige Tian, Hongli Pu, Dongdong Cai, Guangmei Luo, Lili Zhao, Ke Li, Jie Zou, Xiang Zhao, Min Yu, Yayong Wu, Tiankuo Yang, Peng Guo, Xiaoliang Hu

**Affiliations:** 1 Faculty of Agriculture, Forestry and Food Engineering, Yibin Key Laboratory of Zoological Diversity and Ecological Conservation, Yibin University, 644000 Yibin, People’s Republic of China; 2 Aviation Medical Appraisal Center, Civil Aviation Flight University of China, 618307 Guanghan, China; 3 Sichuan Animal Disease Control Central, 610000 Chengdu, People’s Republic of China; 4 College of Veterinary Medicine, Jilin University, 130000 Changchun, People’s Republic of China

**Keywords:** *Japalura sensu lato*, Microbiota, 16S rRNA sequencing analysis, Diversity, Ecology

## Abstract

**Background:**

Gut and oral microbes form complex communities and play key roles in co-evolution with their hosts. However, little is understood about the bacterial community in lizards.

**Results:**

In this study, we investigated the gut and oral bacterial communities in *Japalura sensu lato* from Sichuan Province, China, using 16S rRNA gene sequencing. Results showed that *Bacteroidota* (36.5%) and *Firmicutes* (32.8%) were the main phyla in the gut, while *Proteobacteria*, *Bacteroidota*, *Firmicutes*, and *Actinobacteriota* were the dominant phyla in the oral cavity. 16 S rRNA sequencing analysis of fecal samples showed that: (1) *Bacteroidota* was the most abundant in *Japalura sensu lato*, which was different from the bacterial community of insectivorous animals; (2) *Bacteroidota*, *Firmicutes*, *Actinobacteriota*, *Fusobacteriota*, and *Cyanobacteria* were the most abundant phylum in *Japalura sensu lato*. (3) *Proteobacteria* was the dominant phylum in *Japalura sensu lato* and other domestic insectivorous lizards (*Shinisaurus crocodilurus*, *Phrynocephalus vlangalii*, and *Takydromus septentrionalis*); (4) Comparing with the bacterial community of *Shinisaurus crocodilurus, Phrynocephalus vlangalii, Takydromus septentrionalis, Liolaemus parvus, L. ruibali, and Phymaturus williamsi*, *Desulfobacterota* was uniquely present in the gut of *Japalura sensu lato*. 16 S rRNA sequencing of oral samples showed that *Chloroflexi* and *Deinococcota* phyla were enriched in the oral cavity, which may have a significant influence on living in extreme environments.

**Conclusions:**

Thus, based on 16 S rRNA sequencing analysis of the community composition of the gut and oral microbiomes, this study firstly represents a foundation for understanding the gut and oral microbial ecology of *Japalura sensu lato*, and constitutes a detail account of the diversity of the microbiota inhabiting the gut and oral cavity of *Japalura sensu lato*. Further researches will continue to reveal how gut and oral microbial communities may be impacting the ecology and evolution of lizards.

**Supplementary information:**

The online version contains supplementary material available at 10.1186/s12917-022-03300-w.

## Background

Reptiles are an ancient group containing more than 10 000 species. Over 60% of reptiles belong to the clade Sauria, also known as lizards [1], and exhibit marked diversity in body size, shape, behavior, and life-history strategies [2, 3]. The varied ecological, physiological, and behavioral characteristics of lizards can influence the ecology of their gut and oral microbial communities [4]. However, few investigations have been conducted on the microbial communities of reptiles [5]. The Chinese tree dragon (*Japalura sensu lato*) is primarily distributed in the Yangtze River Basin in southwestern China, including the Yunnan, Sichuan, Chongqing, and Hubei provinces [6]. These lizards often appear on the edge of forests among shrubs and gravel. They are good at climbing, strongly arboreal, highly active, exclusively insectivorous, and usually kept as pets [7].

Vertebrates and invertebrates maintain a complex relationship with their gastrointestinal and oral microbial communities [8, 9]. Gut microbes can affect host behavior [10, 11], immunity [12], nutrition [13] and reproductive isolation [14], ecology, and evolution. To date, the gut microbial communities of nine species of lizards have been reported, including *Liolaemus parvus*, *Liolaemus ruibali*, *Phymaturus williamsi* [15], *Anolis sagrei* [16], *Takydromus septentrionalis* [17], Crocodile Lizards [18], land and marine iguanas [19], *Phrynocephalus vlangalii* [20], *Diploderma vela* [21]. However, two important issues still need to be elucidated: (1) ecology of gut bacterial diversity and (2) how diet, altitude, physiology, and genetics determine microbial population structure [22–24].

Normal oral flora is comprised of various microorganisms, which can be protective and provide an essential barrier through interactions with the host immune system [25]. In addition, oral cavity microbes have co-evolved with their hosts and adapted to diverse conditions for colonization resistance [26]. However, little is understood about the oral bacterial community in lizards.

To expand our understanding of gut and oral microbial diversity in *Japalura sensu lato*, we firstly explored the composition of bacterial communities in the gut and oral cavity using 16 S rRNA sequencing analysis.

## Methods

### Description of samples

The Second Tibetan Plateau Scientific Expedition and Research program included a focus on gut and oral cavity bacterial diversity in reptiles. As such, in the July of 2020 (average temperature 28℃, average humidity 63%), Ten of *Japalura sensu lato* lizards (six females, four males) were 30–33 cm in length and collected from Quebrada in the Laojun Mountains of Sichuan, China, about 110 km from Yibin city (28°84’71’’N; 104°25’30’’E, ~ 600 m above sea level). The lizards, according to the captured time, were named 1–10, respectively and then individually placed in sterilized tubs overnight for the collection of fecal and oral samples using sterile swabs. Each lizard was collected the fecal (named F1-10) and oral (named S1-10) samples respectively. The sterile swabs were placed in RNase-free tubes and transported on dry ice to LE Biotech Co., Ltd. (Shanghai, China). To prevent contamination, the samples were cleaned in advance and none of the sample donors received antibiotic or probiotic therapy. The lizards were released back into the wild after sample collection. We also collected three soil samples (named soil1, soil 2 and soil 3) and foliage from a number of plant species (3 samples, named plant 1, plant 2 and plant 3 for each species of *Oxalis corniculate*, *Setaria viridis, Houttuynia cordata*, *Eremochloa ciliaris, Diplopterygium glaucum*, and *Alsophila spinulosa*), which were collected opportunistically in areas where lizards were captured (< 10 m from point of capture) and pretreated with ethanol-sterilized scissors, placed in RNase-free tubes, and transported on dry ice to LE Biotech Co., Ltd. (Shanghai, China).

### DNA extraction and PCR amplification

The procedures of DNA extraction and PCR amplification were described as previous [27, 28]. Briefly, Microbial DNA was extracted from the fecal and oral samples using the EZNA® Stool DNA Kit (Omega Bio-tek, Norcross, GA, USA) according to the manufacturer’s protocols. The V4-V5 region of the bacterial 16S ribosomal RNA (rRNA) gene was amplified by PCR using primers 515F 5’-barcode-GTGCCAGCMGCCGCGG-3’ and 926R 5’-CCGTCAATTCMTTTRAGTTT-3’, where the barcode is an eight-base sequence unique to each sample [29, 30]. PCR was performed in triplicate in a 20-µL mixture containing 4 µL of 5 × FastPfu Buffer, 2 µL of 2.5 mM dNTPs, 0.8 µL of each primer (5 µM), 0.4 µL of FastPfu Polymerase, and 10 ng of template DNA. Amplicons were extracted from 2% agarose gels and purified using a AxyPrep DNA Gel Extraction Kit (Axygen Biosciences, Union City, CA, USA) according to the manufacturer’s instructions.

### Library Construction and sequencing

As previous described [31], the purified PCR products were quantified by Qubit®3.0 (Life Invitrogen) and every 24 amplicons with different barcodes were mixed equally. The pooled DNA product was used to construct an Illumina paired-end library following the Illumina genomic DNA library preparation procedure. The amplicon library was paired-end sequenced (2 × 250) on an Illumina MiSeq platform (Shanghai BIOZERON Co., Ltd., China) according to standard protocols.

### Processing of sequencing data

Raw fastq files were first demultiplexed using in-house Perl scripts according to the barcode sequence information for each sample with the following criteria: (i) The 250-bp reads were truncated at any site receiving an average quality score < 20 over a 10-bp sliding window, with truncated reads shorter than 50 bp discarded; (ii) exact barcode matching, two nucleotide mismatches in primer matching, and reads containing ambiguous characters were removed; (iii) only sequences with an overlap longer than 10 bp were assembled according to their overlap sequence [31]. Reads that could not be assembled were discarded.

### Statistical analysis

Alpha-diversity (Chao1, Shannon, Simpson, coverage indices) was analyzed using Mothur (v1.35.1) [32] following the protocols of Schloss [33]. The Shannon index and the Chao1 index using normalized OTU table. Principal coordinate analysis (PCoA) based on Bray-Curtis distance metrics was performed in R v3.4.4 to explore the differences in community structures [34]. Comparison across groups were conducted using the adonis function in R on the distance matrices with 999 permutations [35]. Other statistical analyses were performed using SPSS v13.0. Operational taxonomic units (OTUs) were clustered with a 97% similarity cutoff using UPARSE v7.1 (http://drive5.com/uparse/) and chimeric sequences were identified and removed using UCHIME (v4.2.40). The phylogenetic affiliation of each 16 S rRNA gene sequence was analyzed using RDP Classifier (http://rdp.cme.msu.edu/) against the SILVA (SSU132)16 S rRNA database with a confidence threshold of 70% [36]. Redundancy analysis (RDA) was employed to explore the relationship between environmental factors and bacterial communities. Community composition was analyzed at the domain, phylum, class, order, family, and genus levels. For identification of biomarkers for highly dimensional colonic bacteria, LEfSe (linear discriminant analysis effect size) analysis was performed [37]. Kruskal-Wallis sum-rank test was used to examine changes and dissimilarities among classes, followed by local-density approximation (LDA) analysis to determine the size effect of each distinctively abundant taxa [38]. Venn diagrams were drawn using the “Draw Venn Diagram” online tool (http://bioinformatics.psb.ugent.be/webtools/Venn) to analyze overlapping and unique OTUs during the treatment processes.

## Results

### Description of the sequencing data

**Fig. 1:**
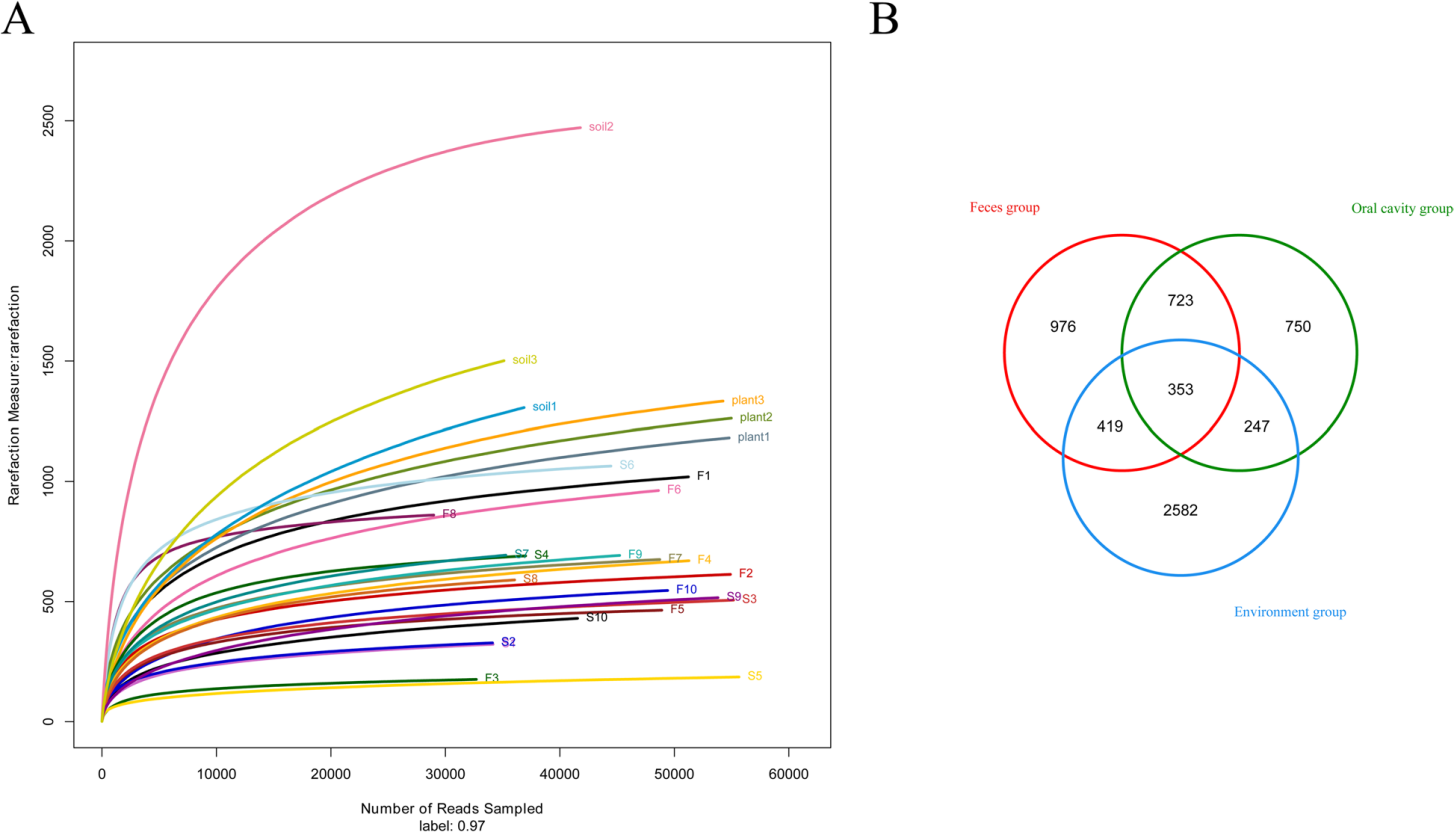
**A** Rarefaction curves of 10 fecal samples (F1–F10), 10 oral samples (S1–S10), three soil samples (soil1–soil3), and three plant samples (plant1–plant3). To evaluate sampling depth, rarefaction curves of microbial communities based on 16 S rRNA gene sequences are shown. **B** Venn diagram of OTUs in feces group, oral cavity group, and environment group

We obtained 1 532 476 raw reads from MiSeq analysis of 26 samples, ranging from 30 470 to 175 731 reads per sample. After read-quality filtering, a total of 1 242 144 quality-filtered reads were obtained, ranging from 29 761 to 59 009 reads per sample, with an average length of 403.58–423.71 bp. A total of 6 156 OTUs were extracted, ranging from 28 982 to 55 646 reads per sample. To compare diversity indices, alpha-diversity (Chao1, Shannon, Simpson, coverage indices), which considers both richness and diversity, was analyzed. The mean coverages of the fecal, oral, and environment groups were 0.997447, 0.9975551, and 0.992444833, respectively (Table 1), indicating that sequencing depth was sufficient to capture the true state of the microorganisms in the samples. The Chao1 estimators were significantly different between the fecal and environment groups (*P* < 0.01) and between the oral and environment groups (*P* < 0.01) but were not statistically different between the fecal and oral groups (Table 1), indicating that OTU richness in the fecal and oral groups was lower than that in the environment groups. The Shannon and Simpson indices were shown that oral groups had a lower community diversity than the fecal and environment groups (Table 1). Rarefaction curves are commonly used to describe the diversity in samples within a group. Here, all curves asymptotically approached a plateau, suggesting that they accurately reflected the microbial community and that the results were sufficient to estimate microbial diversity (Fig. 1A).

**Table 1:** The diversity indices used in this study

Samples(n)	Diversity index					
	Reads	OTU	Chao	Shannon	Coverage	simpson
Feces(10)	45,996.3	667.7	769.7**	4.335	0.997447	0.04371^&^
Oral cavity(10)	42,724.9	532.5	625.4**	3.672*	0.9975551	0.10561^&^
Control(6)	46,300.83333	1,509	1,770.1666	5.0183334	0.992444833	0.047766667

*indicates the values with significant differences between the Fecal group and Control group, and Oral cavity and Control group (*P* < 0.05);

**indicates the values with significant differences between the Fecal group and Control group, and Oral cavity and Control group (*P* < 0.01);

^&^ indicates the values with significant differences between the Fecal group and Oral cavity group (*P* < 0.05);

Based on Venn diagram analysis, 353 OTUs were shared among the fecal, oral, and environment groups. In addition, 976, 750, and 2 582 OTUs were exclusive to the fecal, oral, and environment groups, respectively (Fig. 1B).

**Fig. 2:**
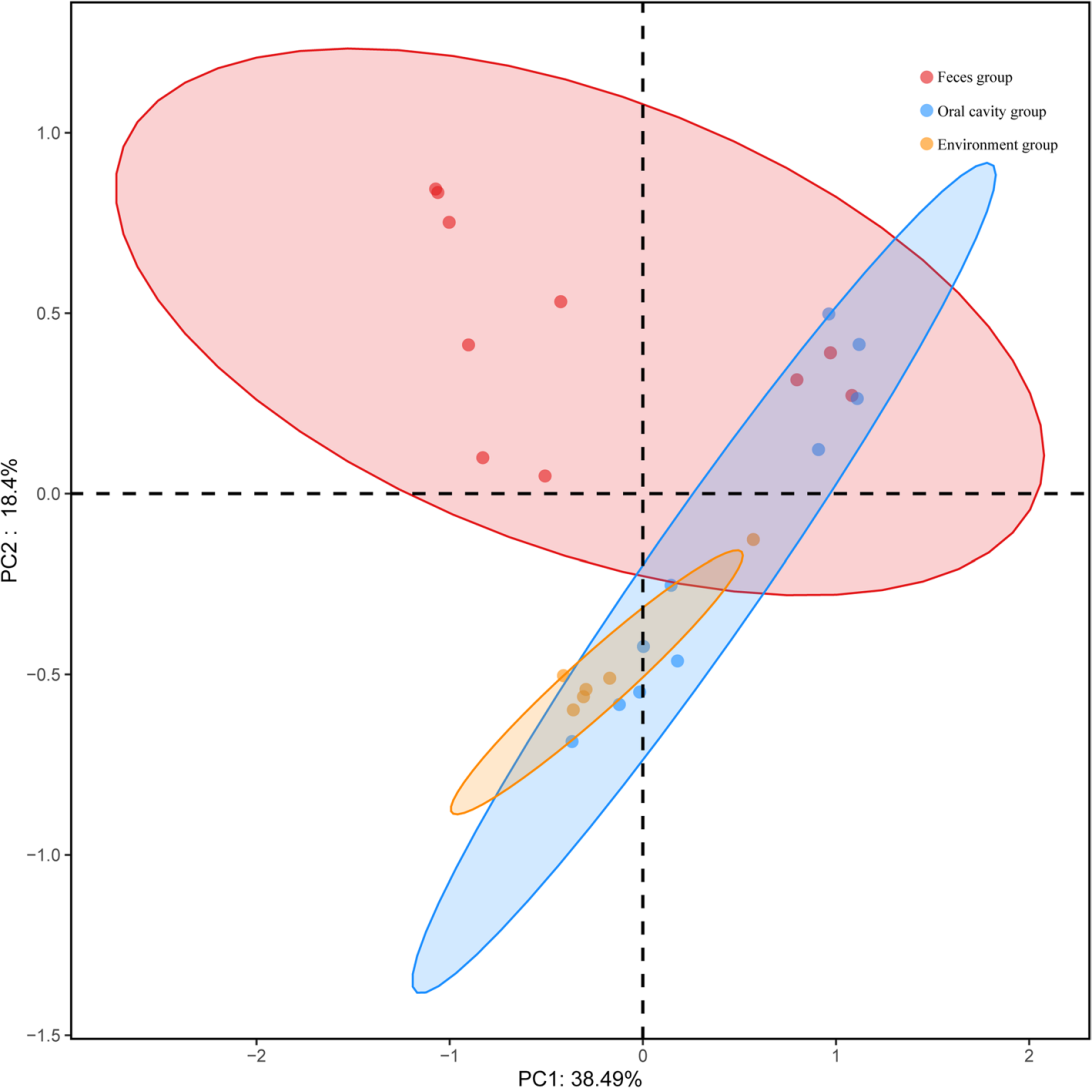
Principal coordinate analysis (PCoA) and clustering analysis, representing dissimilarity in bacterial structure among fecal, oral, and environment (soil and plant) samples. Distances between samples based on OTU composition similarity (OTU similarity ≥ 97%) calculated using unweighted UniFrac distances were visualized by PCoA plots. Percentage of variation explained by PC1 and PC2 are noted on axes

We performed PCoA of overall diversity based on Bray-Curtis distance metrics to compare the microbial diversity of all groups. Analysis showed that there was a significant effect of *Japalura sensu lato* on fecal, oral and environment samples (adonis: feces and oral cavity group, *R*^2^ = 0.17, *P* < 0.01; feces and environment group, *R*^2^ = 0.20, *P* < 0.01; oral cavity and environment group, *R*^2^ = 0.19, *P* < 0.01) (Fig. 2).

### Gut microbial diversity and community composition

**Fig. 3:**
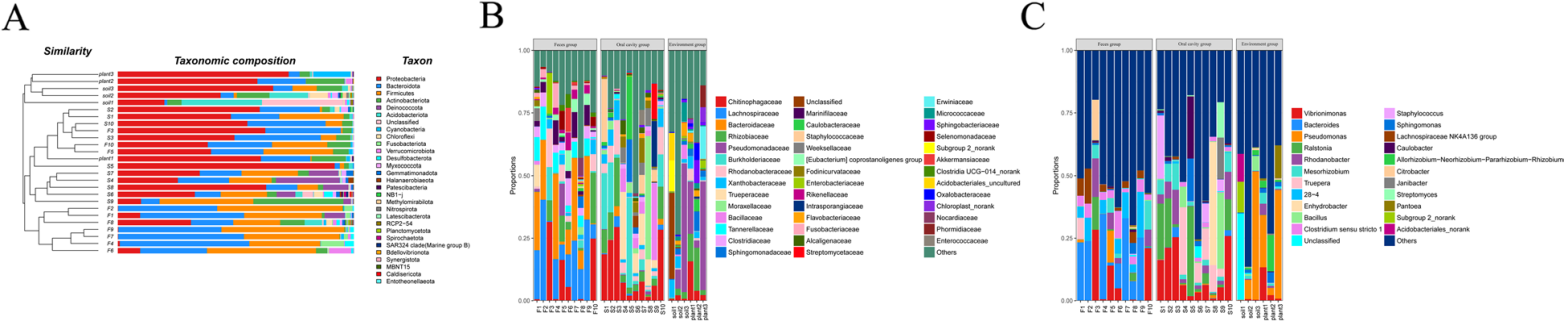
Relative abundance of bacterial communities at phylum (**A**), family (**B**) and genus levels (**C**) in fecal, oral, and environment (soil and plant) samples

The 10 most abundant phyla, families, and genera in the fecal samples are shown in Fig. 3 and Table S1. *Bacteroidota* (36.5%) was the most dominant phylum in the fecal samples, followed by *Firmicutes* (32.8%), *Proteobacteria* (19.1%), *Actinobacteriota* (3.8%), *Fusobacteriota* (1.8%), *Verrucomicrobiota* (1.3%), and *Desulfobacterota* (1.0%), with *Deinococcota* (0.9%), *Acidobacteriota* (0.8%), and *Cyanobacteria* (0.6%) showing relative abundances of < 1.0%.

At the family level, *Lachnospiraceae* (17.0%) and *Bacteroidaceae* (15.8%) were the most dominant in the gut, followed by *Chitinophagaceae* (8.0%), *Tannerellaceae* (5.3%), *Rhizobiaceae* (4.3%), *Marinifilaceae* (4.1%), *Clostridiaceae* (3.9%), and *Rhodanobacteraceae* (3.2%).

At the genus level, *Bacteroides* (15.8%) was the most dominant, followed by *Vibrionimonas* (7.05), *28 − 4* (5.7%), *Parabacteroides* (4.4%), *Clostridium sensu stricto 1* (3.9%), *Lachnospiraceae NK4A136 group* (3.6%), and *Rhodanobacter* (3.1%) (Fig. 3).

For the community composition of fecal and environment samples, seven kinds of bacteria, including *Proteobacteria*, *Actinobacteriota*, *Bacteroidota*, *Acidobacteriota*, *Cyanobacteria*, *Firmicutes* and *Verrucomicrobiota*, were shared in the top 10 dominant phyla.

### Oral cavity microbial diversity and community composition

Bacterial composition in the oral cavity at the phylum level is shown in Fig. 3 and Table S1. Results showed that *Proteobacteria* (47.0%) was the dominant phylum, followed by *Bacteroidota* (18.9%), *Firmicutes* (15.4%), *Actinobacteriota* (6.9%), and *Deinococcota* (6.1%), with *Myxococcota* (0.9%), *Acidobacteriota* (0.8%), *Gemmatimonadota* (0.8%), *Chloroflexi* (0.8%), and unclassified (0.5%) showing relative abundances of < 1.0%.

At the family level, *Chitinophagaceae* (12.9%) was the most dominant, followed by *Burkholderiaceae* (10.0%), *Rhizobiaceae* (6.7%), *Moraxellaceae* (6.2%), *Trueperaceae* (6.1%), *Bacillaceae* (5.2%), *Rhodanobacteraceae* (4.9%), *Staphylococcaceae* (3.8%), *Xanthobacteraceae* (3.3%), and *Caulobacteraceae* (3.0%).

At the genus level, *Vibrionimonas* (11.2%) was the most dominant, followed by *Ralstonia* (9.9%), *Truepera* (6.1%), *Enhydrobacter* (4.9%), *Rhodanobacter* (4.3%), Bacillus (4.1%), *Mesorhizobium* (3.9%), and *Staphylococcus* (3.8%) (Fig. 3).

For the community composition of fecal and oral samples, six types of bacteria, including *Bacteroidota*, *Firmicutes*, *Proteobacteria*, *Actinobacteriota*, *Deinococcota* and *Acidobacteriota*, were shared in the top 10 dominant phyla.

### Comparison of differentially enriched taxa among groups

**Fig. 4:**
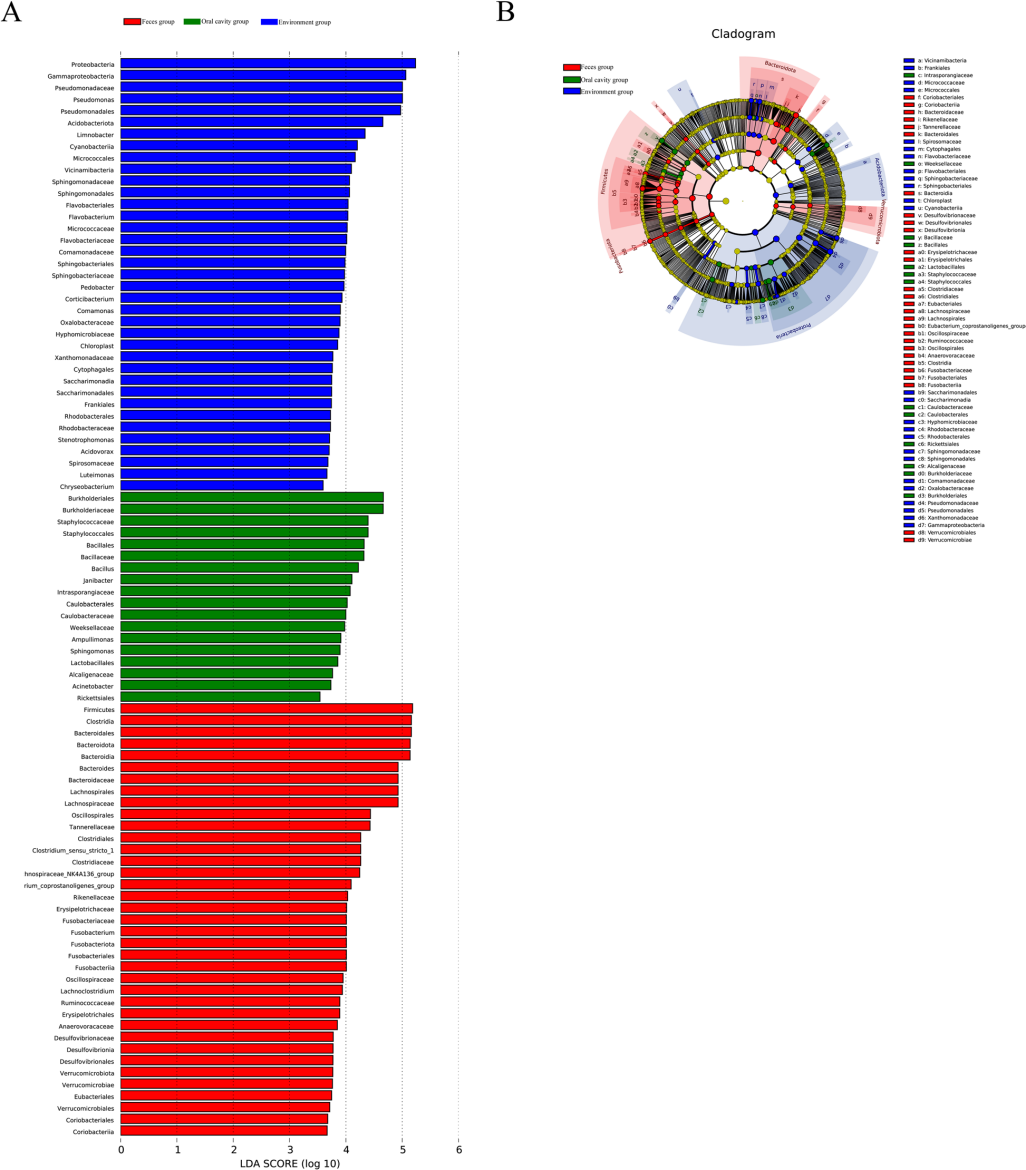
LEfSe (linear discriminant analysis effect size) analysis of microbiota composition of fecal, oral, and environment samples (LDA > 2). **a** Histogram of LDA scores for differentially abundant features in feces group, oral cavity group, and environment group. LEfSe scores were interpreted as degree of consistent difference in relative abundance of microbial communities in fecal, oral, and environment (soil and plant) samples. **b** Taxonomic representation of statistical and biological differences between feces group, oral cavity group and environment group. Differences are represented by colored circles. Color represents classification level and size is proportional to taxon abundance, representing phylum, class, order, and family

Using LEfSe analysis, we selected species showing differences among groups. The results included a LDA distribution histogram, an evolutionary branch diagram (phylogenetic distribution), and an abundance comparison diagram of biomarkers showing statistical differences (LDA score > 2) between groups (Fig. 4). In total, 37 and 18 types of bacteria were enriched in the gut and oral cavity, respectively. *Pseudomonadales (Gammaproteobacteria)*, *Acidobacteriota*, and *Limnobacter* were enriched in the fecal group and played key roles in the microbial community. *Burkholderiales*, *Burkholderiaceae*, *Staphylococcaceae*, *Staphylococcales*, *Bacillales*, *Bacillaceae*, *Bacillus*, *Janibacter* and *Intrasporangiaceae* were enriched in the oral cavity.

## Discussion

According to the diversity and abundance of gut and oral microbiota, many factors, including host species, sex, region, and season, are related to the ecology and behavior of the hosts [15]. A few studies have been performed to examine the differences in gut microbial compositions and abundances in lizards, which suggested that lizards and their microbiota coevolve together [18, 39, 40]. *Japalura sensu lato* is unique to China, whether the gut and oral microbiota is associated with the host habitats and ecology was needed to be determined.

In the present study, *Bacteroidetes* (36.5%) and *Firmicutes* (32.8%) were the dominant phyla found in fecal samples, accounting for 69.3% of sequences, similar to the gut community composition reported in lizard species [19, 20]. Nonetheless, insectivores (*Japalura sensu lato*) (36.5%) and omnivores (*L. parvus* and *L. ruibali*) (35–39%) exhibited higher abundances of *Bacteroidetes* than herbivore (*P. williamsi*) (11–15%) [15]. Although *Bacteroidetes* are abundant in many mammalian gut communities, they show lower abundance in insectivorous mammals such as hedgehogs and house shrews (*Suncus murinus*) [41, 42]. The function of *Bacteroidetes* is to degrade ingested plant-derived material and ferment carbohydrates and short-chain fatty acids [5]. Further research is needed to investigate the role of *Bacteroides* in the insectivorous habit of *Japalura sensu lato.*

*Bacteroidota*, *Firmicutes*, *Actinobacteriota*, *Fusobacteriota*, and *Cyanobacteria* were enriched in *Japalura sensu lato*, *Shinisaurus crocodilurus*, *Phrynocephalus vlangalii*, *Takydromus septentrionalis*, *Liolaemus parvus*, *L. ruibali*, and *Phymaturus williamsi. Proteobacteria* was commonly present in the domestic insectivorous lizards (i.e., *Japalura sensu lato*, *Shinisaurus crocodilurus*, *Phrynocephalus vlangalii*, and *Takydromus septentrionalis*), but absent from the omnivores (*L. parvus* and *L. ruibali*) and herbivores (*P. williamsi*) [15, 17, 18, 20]. *Proteobacteria* can enhance cellulose activity, degrade various aromatic compounds, and promote nutrient absorption in hosts [43]. We found that *Desulfobacterota* was the seventh most abundant phylum in the gut of *Japalura sensu lato* but was absent in the six other lizard species mentioned above. Furthermore, *Desulfobacterota* may be important for sulfate-reducing and fermentative [44, 45]. Based on the above results, we found that gut microbiota abundance and composition were affected by various factors, including geographical region, domestication, diet, and genotype of hosts.

*Firmicutes*, *Actinobacteria*, *Proteobacteria*, and *Bacteroidetes* are the most common phyla found in oropharyngeal samples from various species (e.g., humans, murines, felines, canines, chimpanzees, and hawks) [46–50]. Very few studies have investigated the bacterial composition of the oral cavity in lizards, with research limited to the isolation of bacterial clones from oral and saliva samples using aerobic and anaerobic cultures [51–53] and reports of *Staphylococcus aureus* and *Serratia marcescens* infections in humans following lizard bites [54, 55]. In the current study, we investigated the oral bacterial community in *Japalura sensu lato*. Results showed that *Proteobacteria*, *Bacteroidota*, *Firmicutes*, and *Actinobacteriota* were the dominant phyla in the oral cavity, suggesting similar oropharyngeal bacterial composition as the above hosts. *Chloroflexi* contains anaerobic chemoorganoheterotrophic bacteria with fermentative metabolism in digestive systems [56] and is a dominant phylum in anaerobic wastewater [57]. Thus, *Chloroflexi* was the ninth most abundant phylum in *Japalura sensu lato*, which may contribute to its anaerobic fermentation. The genus *Truepera*, which belongs to the phylum *Deinococcota*, can grow in alkaline, saline, and high temperature environments and is also present in cultivated olives [58, 59]. Our results showed that *Truepera* was a dominant phylum, family, and genus in the oral cavity of *Japalura sensu lato*, which may have high impact on the lizard’s ability to live in extreme environments and regulate the lizard’s body temperature, such as found in southern China with very hot and humid summers. There is another possibility that it is associated with diet, which can shape the microbial community [22].

This study had three main limitations. Firstly, we determined the sex of the lizards, but did not identify their age, which can affect bacterial community composition. Secondly, the samples were collected from one location (Laojun Mountains) and the sample size was small. Thirdly, we did not investigate the influence of season. Thus, our findings should be confirmed using a larger sample size and more collection locations.

## Conclusions

We investigated the composition of the gut and oral bacterial community in an insectivorous lizard species (*Japalura sensu lato*). Our results indicated that *Proteobacteria* was commonly present in domestic insectivorous lizards. *Desulfobacterota* was uniquely present in the gut of *Japalura sensu lato* but was absent in the above six lizard species. *Proteobacteria*, *Bacteroidota*, *Firmicutes*, and *Actinobacteriota* were the dominant phyla in the oral cavity. Furthermore, our study provides new insight into the complex bacterial community and ecology of *Japalura sensu lato* and offers a basic database for further investigations.

## Supplementary Information

**Additional file 1.**

## Data Availability

All 16 S rRNA gene sequences obtained in this study have been deposited in the NCBI Sequence Read Archive under the BioProject accession number PRJNA771761: https://www.ncbi.nlm.nih.gov/sra/PRJNA771761.

## Abbreviations

L. *parvus*: *Liolaemus parvus*
*L. ruibali*: *Liolaemus ruibali*
ORF: Open reading frame
